# Effects and Stress-Relieving Mechanisms of Dark Tea Polysaccharide in Human HaCaT Keratinocytes and SZ95 Sebocytes

**DOI:** 10.3390/molecules28166128

**Published:** 2023-08-18

**Authors:** Chang Gao, Jiafeng Fu, Junyi Cui, Tingzhi Zhang, Christos C. Zouboulis, Jing Wang, Shaowei Yan

**Affiliations:** 1 Syoung Cosmetics Manufacturing Co., Ltd., Changsha 410000, China; hean@syounggroup.com (C.G.); shuren@syounggroup.com (J.F.); dongyi@syounggroup.com (J.C.); dongfang@syounggroup.com (T.Z.); 2 Departments of Dermatology, Venereology, Allergology and Immunology, Staedtisches Klinikum Dessau, Brandenburg Medical School Theodor Fontane, Faculty of Health Sciences Brandenburg, Auenweg 38, 06847 Dessau, Germany; 3 School of Chemistry and Material Engineering, Jiangnan University, Wuxi 214122, China; jingwang@jiangnan.edu.cn

**Keywords:** stress, dark tea polysaccharide, cortisol, sebum

## Abstract

A new skincare application scenario for dark tea, a unique and post-fermented tea popular in the health food industry, was developed in this paper. The effects of dark tea polysaccharide (DTP) on stress-induced skin problems and its mechanism of action were investigated by modeling cortisone-induced stress injury in human HaCaT keratinocytes and SZ95 sebaceous gland cells. The results showed a reduced cortisol conversion induced by cortisone under the action of DTP with a concentration of 200 μg/mL, probably by inhibiting the expression of the HSD11B1 enzyme. DTP was also able to suppress the cortisone-induced elevation of lipid levels in SZ95 sebocytes at this concentration. In addition, the composition and structure of DTP were verified by ultrafiltration, ultraviolet-visible spectrophotometry (UV-VIS), high-performance anion-exchange chromatography with pulsed amperometric detection (HPAEC-PAD) and infrared spectroscopy. In brief, DTP has a unique and significant stress-relieving effect, which provides new ideas for the development of new ingredients for the skin care industry.

## 1. Introduction

Stress is a biochemical and clinical response triggered by environmental or physically external forces. The hypothalamic–pituitary–adrenal (HPA) axis is the main neuroendocrine system involved in stress regulation [1], and its components are interconnected. Faced with stress and adaptive responses, the HPA axis can control the secretion of hormones and stress mediators via feedback regulation, which leads to a series of changes in the physiological activities of the body [2]. In addition, as the largest organ in the body, the skin also has a similar function to the HPA axis with its feedback mechanisms. Skin cells can secrete corticotropin-releasing hormone (CRH), adrenocorticotropin (ACTH) and other hormones. For example, cortisol can be secreted by dermal fibroblasts, epidermal keratinocytes, outer hair root sheath cells, and sebaceous gland cells under the stimulation of CRH and ACTH, which acts as a typical HPA-like axis [3,4]. The skin can also induce epidermal differentiation, pigmentation, vascular response and immune response through endocrine activities regulated by nerves and body fluids to alleviate stress-induced skin reactions, thus maintaining its structural and functional integrity and the balance of its internal environment [5]. However, if the stress response is inadequate or excessive, an overproduction of stress mediators may lead to a range of skin problems such as skin barrier damage, acne, melanin deposition, skin aging and inflammation [3]. Cortisol, an important glucocorticoid, plays a role in regulating the biosynthesis and metabolism of glucose, lipids and proteins, as well as in inhibiting the immune response, and anti-inflammatory, anti-toxic and anti-shock mechanisms [6]. Excessive accumulation of cortisol has been proven to inhibit the proliferation of keratin-forming cells and fibroblasts, thereby preventing the renewal of the skin stratum corneum [7]. It can also weaken the barrier effect of the skin by upregulating the expression of inflammatory factors, which are higher in aging skin [8]. Data from previous studies have shown that cortisol levels are found to be significantly higher in the skin tissue of patients with mental illness or depression than in healthy people; consequently, many studies have used cortisol levels in saliva, hair, or skin to indicate the level of organism-exposed stress [9].

Dark tea is a unique post-fermented tea native to many regions of China including Yunnan, Hunan, Sichuan, and Guangxi provinces [10]. Its manufacturing process generally consists of four major procedures: fixation, rolling, piling and drying, among which the piling is a unique and key procedure in determining the quality of dark tea. The piling procedure involves a series of complex chemical changes in the functional compositions of the tea leaves, which are the origin of the unique color, fragrance and character of dark tea [11]. Tea polysaccharide is an acidic glycoprotein, a class of complexes composed of sugars, pectins and proteins in tea leaves that contain a number of mineral elements. During the fermentation processing and storage of dark tea, microorganisms contained within the plants can secrete a large amount of cellulase, which decomposes the cellulose to produce water-soluble polysaccharide, leading to higher water-soluble polysaccharide content in dark tea than in teas such as green tea and black tea [12]. In addition, dark tea polysaccharides are hydrolyzed into peptide chains with short molecular chains, resulting in easy absorption and strong pharmacological activity under the combined action of enzymes secreted by microorganisms. Hence, due to its unique components, dark tea has certain pharmacological effects, such as fat reduction [13], anti-oxidant [14,15,16], anti-tumor [17,18], immunomodulatory [19] and anti-fatigue [20]. Although dark tea is widely known in the healthcare industry because of its unique pharmacological effects, its application in the skincare industry has rarely been reported. Therefore, in this study, dark tea polysaccharide (DTP) was prepared from dark tea, and its inhibitory effect on cortisol in human HaCaT keratinocytes and the corresponding mechanism, as well as on stress-induced lipid secretion in human SZ95 sebocytes, were investigated by molecular biology and biochemistry. The composition and structure of DTP were also analyzed by UV-vis, infrared spectroscopy and other techniques. Our results show that DTP can alleviate skin problems caused by stress and provide a theoretical basis for the application of DTP in combating stress.

## 2. Results

### 2.1. Analysis of the DTP Molecular Weight Composition

As shown in Table 1, DTP molecules with a molecular weight of more than 100 KD accounted for about 50% of the mass of the extracted DTP. Among the DTP molecules with a molecular weight of less than 100 KD, those with a molecular weight of less than 10 KD accounted for a larger proportion—about 36% in total—while those with a molecular weight of 10–100 KD accounted for the lowest proportion—about 14% in total.

### 2.2. Analysis of the DTP Chemical Composition

In this study, four major chemical components, namely total sugar, total protein, total polyphenols and uronic acid, were quantitatively analyzed using UV-Vis (Table 2).

### 2.3. Analysis of the DTP Monosaccharide Composition

**Figure 1:**
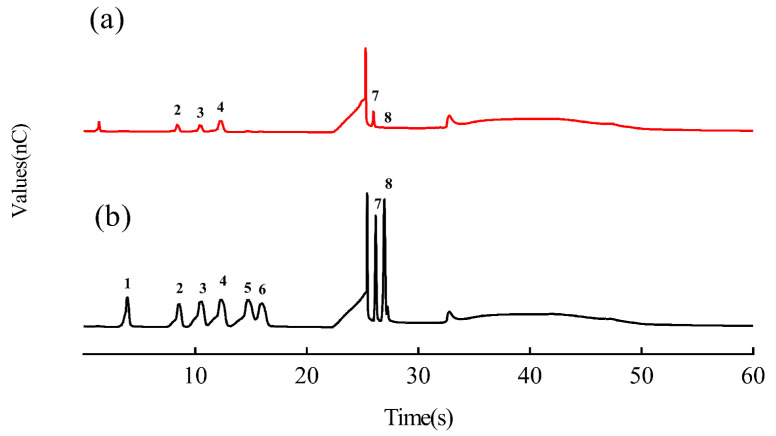
High-performance anion-exchange chromatography with pulsed amperometric detection (HPAEC-PAD) profiles of DTP (**a**) and mixed standards (**b**). The numbers in the profiles represent the following: 1. Fucose; 2. Arabinose; 3. Galactose; 4. Glucose; 5. Xylose; 6. Mannose; 7. Galacturonic acid; 8. Glucuronic acid.

Figure 1 shows high-performance anion-exchange chromatography with pulsed amperometric detection (HPAEC-PAD) profiles of DTP samples and mixed standards. The samples had response times (Figure 1a) of 8.4 min, 10.5 min, 12.3 min, 25.9 min and 26.8 min. Based on the response times of the standards (Figure 1b), it was determined that the samples mainly contained arabinose, galactose, glucose, galacturonic acid and glucuronic acid. According to the quantitative analysis of the peak areas of the standards (Table 3), the molar ratios of arabinose, galactose, glucose, galacturonic acid and glucuronic acid were about 1:1:2:1.8:0.3.

### 2.4. Analysis of DTP Structural Features

**Figure 2:**
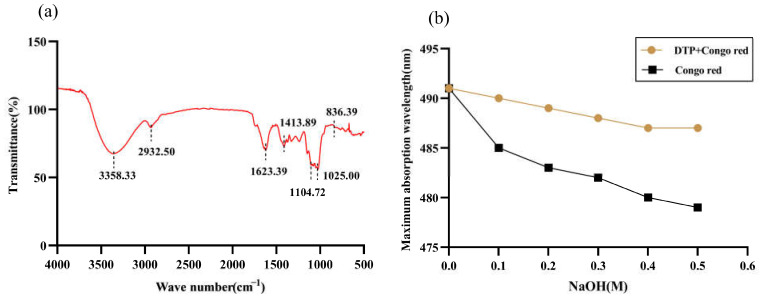
Infrared spectral analysis of DTP (**a**); Maximum absorption wavelengths of Congo red with DTP at different NaOH concentrations (**b**).

Figure 2a shows the infrared spectrum of DTP. The broad peak at 3358.33 cm^−1^ corresponds to the stretching vibration peak of O-H and the absorption peak at 2932.50 cm^−1^ corresponds to the stretching vibration peak of C-H, both of which are characteristic absorption peaks of sugars. In addition, the peak of 1623.39 cm^−1^ corresponds to the bending vibration of O-H and the peak of 1413.89 cm^−1^ corresponds to the variable angle vibration of C-H, which are characteristic absorption peaks of uronic acid. The peaks at 1104.72 cm^−1^ and 1025.00 cm^−1^ are the characteristic peaks of the pyran ring and correspond to the stretching vibrations of C-O on the pyran ring. The peak at 836.39 cm^−1^ is the characteristic absorption peak of the α-glycosidic bond. There are also some small absorption peaks between 900 and 1000 cm^−1^, which are the characteristic absorption peaks of the β-glycosidic bond.

The changes in the maximum absorption wavelength of Congo red complexed with DTP under different mass concentrations of NaOH solution are shown in Figure 2b. The maximum absorption wavelength of DTP mixed with Congo red solution showed a similar trend to that of Congo red solution with increasing NaOH, i.e., in both cases, the wavelength decreased gradually. This shows that DTP does not have a triple-helix conformation.

### 2.5. Effect of DTP on Viability of HaCaT Keratinocyte Cells

**Figure 3:**
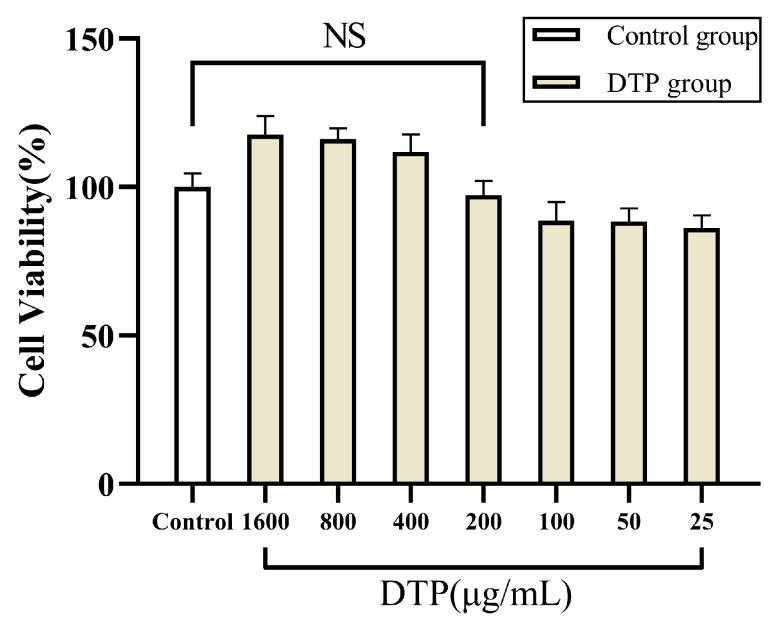
Cell viability of HaCaT keratinocytes co-incubated with DTP at different concentrations (NS: No Significance, *n* ≥ 3).

The results of MTT experiments suggested that there was no significant change in the cell viability of HaCaT keratinocytes in a 200 μg/mL solution of DTP, retaining 97.27% of the cell viability of the control group (Figure 3). However, the cell viability decreased to only 88.69% of that of the control group when the concentration of DTP was reduced to 100 μg/mL. Therefore, DTP with a concentration of 200 μg/mL was chosen for the subsequent experiments.

### 2.6. Cortisol Production in HaCaT Keratinocytes after DTP Treatment

**Figure 4:**
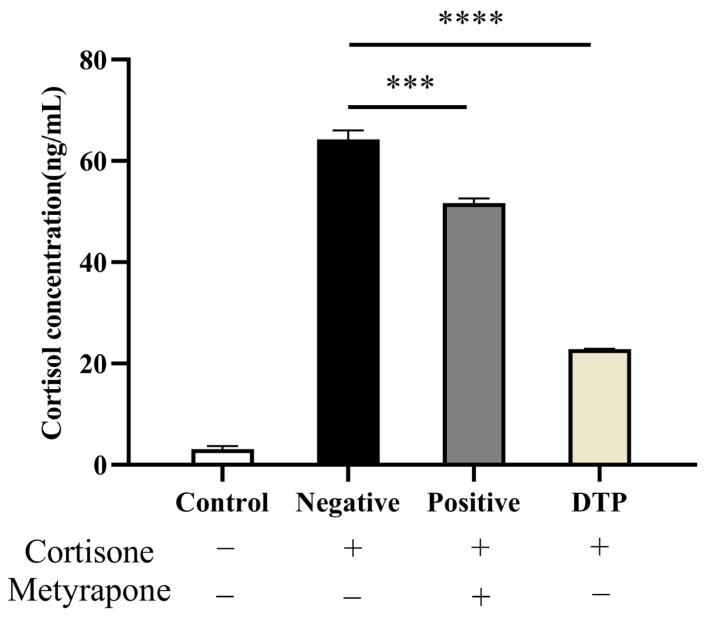
Inhibitory effects of DTP on cortisol with a cortisone concentration of 100 μmol/L, Metyrapone concentration of 200 μmol/L and DTP concentration of 200 μg/mL (*** *p <* 0.001, **** *p <* 0.0001, *n* ≥ 3).

The cortisol content was inferred from the absorbance of the standard curve, and the results are shown in Figure 4. The basal secretion of untreated HaCaT keratinocytes (ELISA) was 3.0945 ng/mL. As for the negative group, cortisone at 100 μmol/L concentration greatly promoted cortisol secretion into the medium. Through the intervention of Metyrapone and DTP, the cortisol level of the positive administration group and DTP group was suppressed significantly, especially in the case of the DTP group.

### 2.7. Effect of DTP on HSD11B1 Enzyme Expression

**Figure 5:**
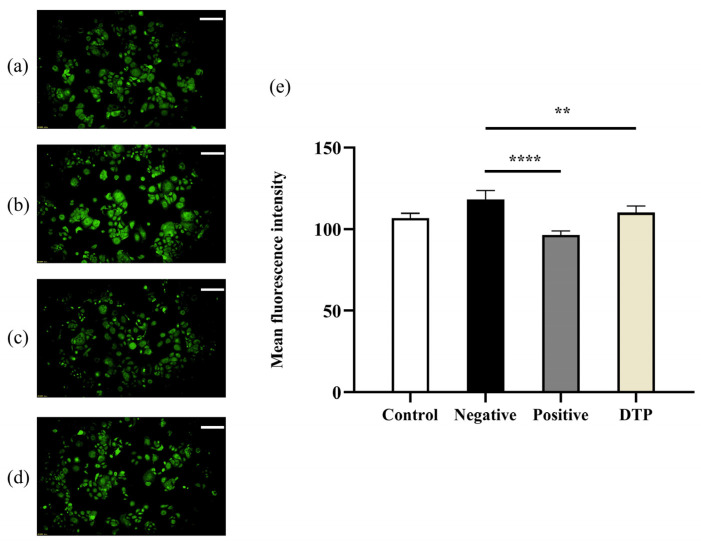
Inhibition of HSD11B1 enzyme expression by DTP. (**a**) Untreated control group; (**b**) With the 24 h intervention of cortisone at a concentration of 100 μmol/L; (**c**) With cortisone and the inhibitor Metyrapone (200 μmol/L) added simultaneously; (**d**) With cortisone and DTP (200 μg/mL) added simultaneously; (**e**) Statistical analysis of the HSD11B1 mean fluorescence intensity (Scale Bar: 100 μm; ** *p* < 0.01, **** *p <* 0.0001, *n* = 3).

HSD11B1 is the key enzyme for the conversion of inactive cortisone to active cortisol. Immunofluorescence experiments showed that the mean fluorescence intensity of HSD11B1 was weak in the untreated control group (Figure 5a) and was significantly enhanced by the addition of cortisone (Figure 5b), while the mean fluorescence intensity of HSD11B1 was weak when the inhibitor Metyrapone (Figure 5c) or DTP (Figure 5d) was added simultaneously. A statistical analysis with significant differences (Figure 5e) suggested that DTP could inhibit the expression levels of HSD11B1 enzyme induced by cortisone.

### 2.8. Effect of DTP on Viability of SZ95 Sebocytes

**Figure 6:**
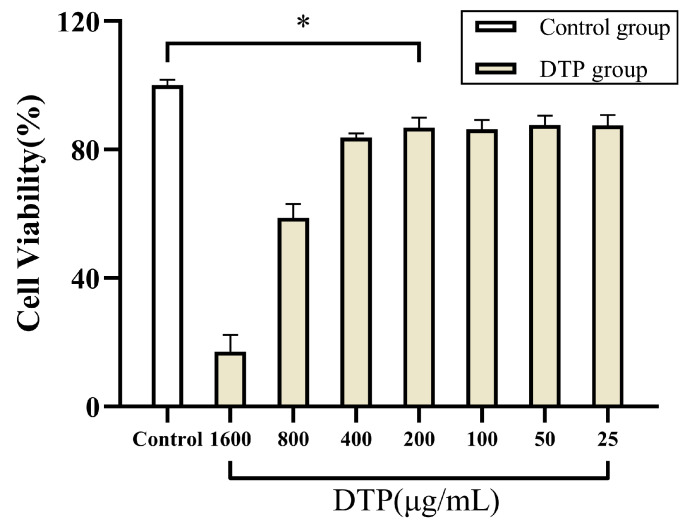
Cell viability of SZ95 sebocytes co-incubated with DTP at different concentrations (* *p* < 0.05, *n* ≥ 3).

The results of MTT experiments suggested that the cell viabilities of SZ95 sebocytes in the presence of DTP were significantly different from that of the control group at all concentrations of DTP, but the cell viability values at DTP concentrations of 25, 50, 100 and 200 μg/mL were closer to that of the control group (Figure 6). Based on these results and the results of the MTT experiments on HaCaT keratinocytes, DTP with a concentration of 200 μg/mL was finally chosen for the subsequent experiments.

### 2.9. Cortisol Production and Lipid Secretion in SZ95 Sebocytes after Cortisone Treatment

**Figure 7:**
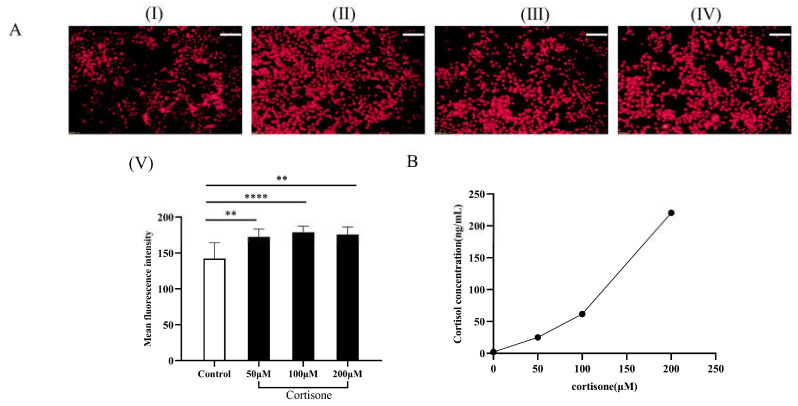
Promotion effects of cortisone on lipid secretion and cortisol levels. (**A**) (I) Untreated control group; With 24 h intervention of cortisone at concentrations of 50 μmol/L (II); 100 μmol/L (III); and 200 μmol/L (IV); (V) Statistical analysis of the experimental results of each group; (**B**) Cortisol levels in the supernatant of SZ95 sebocytes (Scale Bar:100 μm; ** *p <* 0.01, **** *p <* 0.0001, *n* = 3).

The skin tends to produce more lipids in stressful situations. The lipid content of SZ95 sebocytes in the presence of cortisone was assessed using Nile red staining (Figure 7). It can be seen that cortisone could stimulate SZ95 sebocytes to secrete more lipids in a dose-dependent manner (Figure 7A). Determining the levels of cortisol in the supernatant of SZ95 sebocytes by ELISA assay showed that there was a positive correlation between cortisol levels and increasing cortisone concentration (Figure 7B). The results show that cortisone can induce an increase in cortisol content in SZ95 sebocytes and significantly activate cellular lipid-secreting activity.

### 2.10. Effect of DTP on Lipid Secretion Induced by Cortisone

**Figure 8:**
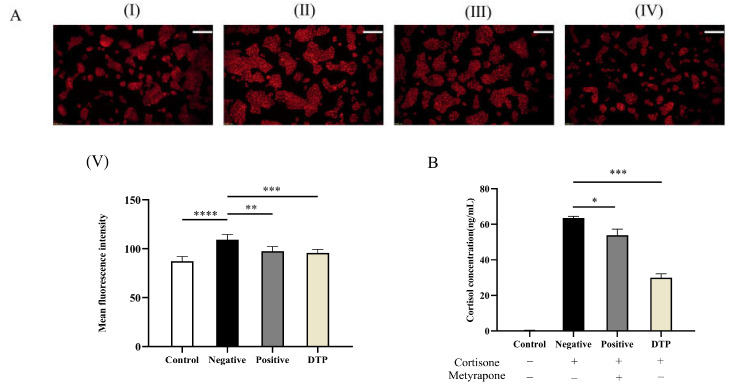
Inhibitory effects of DTP on lipid secretion and cortisol. (**A**) (I) Untreated control group; With the 24 h intervention of cortisone at a concentration of 100 μmol/L (II); cortisone and the inhibitor Metyrapone (200 μmol/L added simultaneously (III); and cortisone and dark tea polysaccharide (200 μg/mL) added simultaneously (IV); (V) Statistical analysis of the experimental results of each group; (**B**) The levels of cortisol in the supernatant of SZ95 sebocytes with a cortisone concentration of 100 μmol/L, a Metyrapone concentration of 200 μmol/L and a DTP concentration of 200 μg/mL (Scale Bar: 100 μm; * *p <* 0.05, ** *p <* 0.01, *** *p <* 0.001, **** *p <* 0.0001, *n* = 3).

Next, the inhibitory effects of DTP and the inhibitor Metyrapone on lipid secretion and cortisol levels in SZ95 sebocytes were investigated. SZ95 sebocytes were stained with Nile Red to evaluate the lipid content in the cells, and the cell supernatant was collected simultaneously for ELISA assay to determine the cortisol content. The oil fluorescence intensities of the DTP and inhibitor groups were significantly weakened compared to the control group (Figure 8A). Meanwhile, the ELISA assay suggested that DTP could inhibit cortisol conversion induced by cortisone (Figure 8B). In view of the above results, it was inferred that DTP could reverse the increase in lipid levels in SZ95 sebocytes induced by cortisone.

## 3. Discussion

The skin can secrete a variety of HPA-axis-related hormones, with the main mediators including pro-opiomelanocortin (POMC), ACTH, CRH and cortisol [4]. HPA axis secretory activity increases and cortisol secretion elevates under stressful conditions including prolonged exposure to environmental or emotional stress, resulting in cortisol levels in skin tissue and skin appendages becoming significantly elevated [21]. In brief, stress can cause an overproduction of cortisol, leading to a range of skin disorders [7,8]. Therefore, the ability of materials to inhibit the conversion of cortisone to cortisol has been used as an important indicator to assess the ability of the skin to counteract stress damage. In this study, we used cortisone to stimulate HaCaT keratinocytes and construct a stress evaluation model and found that cortisone could promote cortisol secretion from HaCaT keratinocytes while DTP could significantly inhibit cortisol conversion.

Cortisone and cortisol (hydrocortisone) are presentative substances of natural glucocorticoids. Approximately 95% of cortisone is bound to cortisone-binding globulin (CBG, also known as corticosteroid transport protein) in an inactive state and needs to be transported into cells to bind to glucocorticoid receptors and form a complex that affects the transcriptional activation or repression of genes [22,23]. The cholesterol side chain cleavage enzyme CYP11A1 can catalyze the conversion of cholesterol to the C21 steroid pregnenolone, which is then converted to cortisone through a series of enzymatic reactions. The 11β-steroid dehydrogenase 1 (HSD11B1) catalyzes the production of cortisol. The regeneration and metabolism of cortisol are mainly regulated by HSD11B1 to exert a series of biological effects [24,25]. HSD11B1 can convert inactive cortisone into active cortisol with the participation of NADPH, and therefore, the expression of HSD11B1 determines the bioavailability of cortisol [26]. To further investigate the mechanism of action of DTP in inhibiting cortisol conversion, the effect of DTP on HSD11B1 expression was investigated. In studying the effect of DTP on HaCaT keratinocyte and SZ95 sebocyte cell viability, it was found that DTP with a concentration of 200 μg/mL did not exhibit cell toxicity for either cell line, so a concentration of 200 μg/mL DTP was selected for the subsequent experiments. The follow-up study showed that DTP could significantly inhibit the expression of HSD11B1 enzyme induced by cortisone; thus, it could be inferred that DTP could inhibit the conversion of cortisol by inhibiting the expression of HSD11B1 enzyme.

Under stressful conditions, the central HPA axis and the cutaneous HPA axis are activated and stress hormones such as CRH and ACTH are released, activating 3β-hydroxysteroid dehydrogenase in sebaceous gland cells. 3β-hydroxysteroid dehydrogenase converts dehydroepiandrosterone into androgens, promoting sebaceous gland proliferation and differentiation and lipid secretion [27,28,29]. In the present study, a cortisone-induced sebum secretion model in SZ95 sebocytes was used to simulate the effect of a stressful environment on skin sebum secretion. It was found that cortisol levels in sebaceous gland cells were significantly elevated after a cortisone-induced intervention, while sebaceous gland cells secreted more lipids. This result suggested that skin lipid secretion was higher under stressful conditions than under non-stressed conditions. Further investigation of the effect of DTP on cortisol levels and lipid secretion in SZ95 sebocytes revealed that DTP could inhibit cortisol levels while reversing the cortisone-induced elevation of lipid levels in SZ95 sebocytes. Therefore, it could be inferred that DTP can alleviate the oiliness of skin caused by stress. Whether cortisol promotes lipid overproduction by acting on 3β-hydroxysteroid dehydrogenase in sebaceous gland cells remains to be demonstrated.

Dark tea is popular in the healthcare industry due to its wide range of pharmacological activities but there are still relatively few studies on DTP. Therefore, DTP containing molecules of different molecular weights was collected using ultrafiltration in this paper. The smaller molecular weight fraction of the DTP accounted for a higher percentage, which may be due to the microorganisms present in dark tea and the unique fermentation process promoting the hydrolysis of large polysaccharides into small polysaccharides with higher activity [12]. Experiments on the chemical composition of DTP using UV-Vis spectroscopy showed that DTP had a relatively high content of total sugars and uronic acid alongside small amounts of polyphenolic and protein components. An HPAEC-PAD investigation showed that the main monosaccharides in DTP are arabinose, galactose, glucose, galacturonic acid and glucuronic acid in a molar ratio of about 1:1:2:1.8:0.3. This indicates that DTP is an acidic polysaccharide. Acidic polysaccharides usually have a high biological activity as well as an ability to regulate human immunity [19]. In the present study, DTP was found to be an acidic polysaccharide with α and β glycosidic bonds and a high percentage of small molecular weight polysaccharides and soluble polysaccharides, which provided evidence for the origin of the stress-relieving effects of DTP. Finally, while the stress evaluation in this study was conducted using skin cells, the authors hope that subsequent studies could validate the results on skin models and clinical trials to provide more evidence for the efficacy of the application of this ingredient in cosmetics.

## 4. Materials and Methods

### 4.1. DTP Preparation

The dark tea used in this paper was obtained from the Baishachong tea factory (Anhua County, Yiyang City, Hunan Province, China). After drying in the oven at 50 °C for 20 min, the dark tea was crushed and passed through an 80-mesh sieve to obtain dark tea powder. A certain mass of black tea powder was weighed according to a solid–liquid ratio of 1:25, extracted in an oil bath at 80 °C for 60 min and filtered to obtain the filtrate for use. The tea residue after filtration was repeatedly extracted according to the above conditions, and then the two filtrates were combined and concentrated to 100 mL under reduced pressure at 60 °C. A 300–400 mL volume of ethanol was added to the dark tea concentrate, and then the solution was refrigerated at 4 °C after thorough stirring. The precipitate was collected by centrifugation (8000× *g* rpm, 6 min) after 24 h and washed twice with ethanol. Finally, the precipitate was freeze-dried for 72 h to obtain DTP powder.

### 4.2. Analysis of the Molecular Weight Composition in DTP Using Ultrafiltration

The DTP prepared in 4.1 was dissolved with water and then the solution was centrifuged (3800× *g* rpm, 30 min) through an ultrafiltration centrifuge tube with a molecular weight cutoff of 100 KD to collect the polysaccharide retention solution with a molecular weight of greater than 100 KD. Similarly, the permeate was collected and then passed through an ultrafiltration centrifuge tube with a molecular weight cutoff of 30 KD to collect polysaccharides with molecular weights of between 30–100 KD. Finally, the permeate was passed through an ultrafiltration centrifuge tube with a molecular weight cutoff of 10 KD to obtain polysaccharides with molecular weights of less than 10 KD and 10–30 KD. The obtained DTP fractions with different molecular weights were lyophilized and weighed for analysis.

### 4.3. Analysis of the Chemical Composition in DTP Using UV-VIS

The total sugar contents in DTP were determined by the Anthrone-sulfate method [30] using glucose as a standard. Bovine serum albumin (BSA) was used as a standard for the determination of protein content in DTP by the Coomassie brilliant blue method [31]. The polyphenol content in DTP was determined by the Folin–Ciocalteu method [32] using gallic acid as a standard. The carbazole colorimetric method [33] was used to determine the glucuronic acid content in DTP with glucuronic acid as a standard.

### 4.4. Analysis of the Monosaccharide Composition in DTP Using HPAEC-PAD

The monosaccharide composition of DTP was analyzed by high-performance anion-exchange chromatography with pulsed amperometric detection (HPAEC-PAD). An appropriate amount of the sample prepared in 4.1 was weighed and 0.5 mL of 12 mol/L sulfuric acid was added in an ice bath. Then, the solution was stirred for 30 min at room temperature, diluted with water and left to hydrolyze for 2 h. After cooling, the sample was diluted across the membrane for analysis. Chromatographic column: CarboPacTMPA20 (3 × 150 mm); working electrode: Au; reference electrode: Ag/AgCl; mobile phase A-deionized water, B-25 mmol/L NaOH solution, C-1 mol/L NaOAc solution, D-200 mmol/L NaOH solution.

### 4.5. Infrared Spectral Analysis of DTP

The DTP prepared in 4.1 was analyzed in an FTIR Spectrometer using the potassium bromide pellet technique scanning from 500 cm^−1^ to 4000 cm^−1^.

### 4.6. Congo Red Experiment of DTP

A 1 mL volume of DTP solution (100 μg/mL) was placed in a test tube followed by 1 mL of Congo red [34] working solution (200 μg/mL) and then the two were fully mixed. Subsequently, an appropriate amount of NaOH (10 M) mother liquor was added to create the following concentration gradient: 0 M, 0.1 M, 0.2 M, 0.3 M, 0.4 M, 0.5 M. Finally, the absorption of the solutions at 300–600 nm was measured using a UV spectrophotometer.

### 4.7. Cell Culture

Human HaCaT keratinocytes were maintained at 37 °C and 5% carbon dioxide in MEM medium (Procell, Wuhan, China) supplemented with a 1% antibiotic mixture in the presence of 15% FBS (Gibco, New York, NY, USA). Human SZ95 sebocytes [35] were maintained at 37 °C and 5% carbon dioxide in DMEM medium (Gibco, New York, NY, USA) supplemented with a 1% antibiotic mixture in the presence of 10% FBS (Gibco, New York, NY, USA).

### 4.8. MTT Assay

The cells were treated with DTP for 24 h and the cell viability was detected. Under lightproof conditions, 10 μL of MTT solution (5 mg/mL) was added to each well of a 96-well plate, and then the plate was placed in an incubator at 37 °C with 5% CO_2_ for 4 h. Subsequently, the supernatant in the plate was removed, 150 μL/well of DMSO was added to the residue and then the residue was left to dissolve for 10 min. The absorbance at 570 nm of each well was detected using an enzyme standardization instrument with 630 nm as a reference wavelength.

### 4.9. ELISA

The supernatant from each well plate was collected to detect the cortisol content by ELISA assay with the following steps. The standard was diluted to 200, 100, 50, 12.5 and 0 ng/mL with sample. A 50 μL volume of standard working solution and assay sample was added to each reaction well with two replicate wells for each group. Next, 50 μL of biotin-labeled antibody working solution was added to each reaction well to give a 100 μL reaction system, and the wells were incubated at 37 °C for 45 min. Subsequently, the solution was left to soak for 1–2 min and then the excess fluid was shaken off. A 100 μL volume of HRP-labeled streptavidin working solution was added to each well and the wells were incubated at 37 °C for 30 min. The washing solution was left to soak and was then shaken off. A 90 μL volume of color developer was added to each well and then the wells were incubated at 37 °C for 15 min. Finally, a 50 μL volume of termination solution was added to each reaction well and the OD value was measured immediately at 450 nm with an enzyme marker. Cortisol concentration was calculated on the basis of the standard curve regression equation (R^2^ > 0.99).

### 4.10. Experiment of Cortisol Inhibition in HaCaT Keratinocytes

HaCaT keratinocytes in good growth condition were made into a suspension and adjusted to a density of 2 × 10^6^ cells/mL. The suspension was inoculated into 96-well plates (100 μL/well) and incubated at 37 °C in 5% CO_2_. Blank control, negative control, positive control and sample (DTH) groups were each set up. The cells were intervened with 100 μmol/L of cortisone for 24 h (in the case of the negative control group, an equal amount of medium was added). Finally, the supernatant was taken from each well plate and cortisol levels were measured by ELISA.

### 4.11. Experiment on the Change of HSD11B1 Enzyme Expression in HaCaT Keratinocytes

HaCaT keratinocyte suspension adjusted to a density of 2 × 10^6^ cells/mL was inoculated into 96-well plates (100 μL/well) and incubated at 37 °C in 5% CO_2_. Blank control, negative control, positive control and sample (DTH) groups were once again set up. Cells were pretreated for 4 h and then intervened with 100 μmol/L cortisone for 24 h (in the case of the blank control group, an equal amount of medium was added). Then, the cells were washed with phosphate-buffered solution (PBS) and fixed with 4% paraformaldehyde solution. The cytoplasmic membrane was permeabilized with 0.1% Triton X-100. Primary antibody HSD11B1 polyclonal antibody was incubated overnight at 4 °C and then FITC fluorescently labeled secondary antibody was added to completely cover the cells, which were then incubated for 1 h at room temperature, protected from light. Photographs were taken under a fluorescent microscope.

### 4.12. Experiment on Cortisol Conversion/Lipid Secretion Assay Induced by Cortisone in Sebaceous Gland Cells

Human SZ95 sebocytes were maintained at 37 °C and 5% carbon dioxide in DMEM medium (Gibco, New York, NY, USA) supplemented with antibiotic mixture in the presence of 10% FBS (Gibco, New York, NY, USA). The experiment was performed in two parts simultaneously, with the cell supernatant being collected to assay the cortisol content while the cells were collected for Nile Red staining to assay the cytosolic lipid production. The experiments were grouped into different concentrations of cortisone treatment groups: 50 μmol/L, 100 μmol/L and 200 μmol/L. The control group was incubated for the same amount of time with equal amounts of medium.

### 4.13. Experiment on Lipid Secretion Induced by Cortisone with DTP in Sebaceous Gland Cells

The negative control group was treated with a 100 μmol/L concentration of cortisone for 24 h. The positive control and DTP groups were also treated with 200 μg/mL Metyrapone or 200 μmol/L DTP under this condition. The control group was incubated for the same duration by adding an equal amount of medium. Images were analyzed with Image J, and graphs were analyzed with GraphPad Prism 8.0.

### 4.14. Image J Fluorescence Intensity Analysis

All fluorescence images were captured using a fluorescence microscope with 10× objective lenses (OLYMPUS IX73). The mean intensity of fluorescence was processed by ImageJ software(Image J 1.53k, Wayne Rasband, Bethesda, MD, USA). First, split channels were used to divide each fluorescent image into red, green and blue images. The green fluorescent image was retained for analysis and the other two images were set aside. Next, the threshold was adjusted and the appropriate area was selected, and then the threshold was represented in red. Subsequently, analysis was performed, and the average gray intensity was taken to represent the average fluorescence intensity of the green fluorescence image.

### 4.15. Statistical Analysis

GraphPad Prism 8.0 statistics were used (Mean ± SEM). Two groups were compared by independent samples *t*-test and multiple groups were compared by one-way ANOVA (one-way ANOVA), and *p* < 0.05 was considered statistically significant.

## 5. Conclusions

Dark tea is a natural product widely used in food and pharmaceutical applications. However, in this paper, we validated for the first time the unique skin stress-relieving action and mechanism of dark tea polysaccharide and determined its unique characteristic composition at the molecular level. It was found that DTP (200 μg/mL) could inhibit the conversion of stress-mediated cortisol, probably by inhibiting the expression of HSD11B1 enzyme. DTP could also reverse the elevation in the level of lipids induced by cortisone in SZ95 sebocytes. Meanwhile, composition tests showed that DTP is an acidic polysaccharide with α and β glycosidic bonds and contains a high percentage of small molecular weight polysaccharides and soluble polysaccharides. The findings suggest that DTP has a strong stress-relieving effect, and provide a theoretical basis for the application of dark tea in cosmetics, especially in solving a host of skin problems caused by stress. However, whether cortisol plays a role in promoting lipid overproduction by activating 3β-hydroxysteroid dehydrogenase in sebaceous gland cells needs to be further verified. In order to advance the application of DTP in the skincare industry, subsequent studies could validate these findings on skin models and in clinical trials.

## Figures and Tables

**Table 1:** The mass distribution of polysaccharides in DTP.

Molecular Weight	Mass Ratio
<10 KD	36.13%
10–30 KD	7.92%
30–100 KD	5.94%
>100 KD	50.01%

**Table 2:** Quantitative linear ranges, quantitative analysis results of the chemical composition of DTP.

No.1	Components	Regression Equation	Composition (mg/g)
1	Total sugar	Y = 0.0036X + 0.0014 (R^2^ = 0.9991)	509.13
2	Total protein	Y = 0.0037X + 0.0384 (R^2^ = 0.9952)	57.17
3	Total polyphenols	Y = 0.0050X + 0.0102 (R^2^ = 0.9985)	49.84
4	Uronic acid	Y = 0.0104X +0.2597 (R^2^ = 0.9926)	101.42

**Table 3:** Quantitative linear ranges, correlation coefficients (R^2^) and retention times (RT) of the HPAEC-PAD standards.

No.1	Components	Regression Equation	Range (μg/mL)	RT (min)
1	Fucose	Y = 1.3322X + 3.9023 (R^2^ = 0.9992)	2.5–12.5	3.9
2	Arabinose	Y = 1.5408X + 3.0415 (R^2^ = 0.9995)	2.5–12.5	8.4
3	Galactose	Y = 2.3057X + 5.0154 (R^2^ = 0.9998)	2.5–12.5	10.5
4	Glucose	Y = 2.8522X + 5.3423 (R^2^ = 0.9991)	2.5–12.5	12.3
5	Xylose	Y = 3.3568X + 4.6115 (R^2^ = 0.9998)	2.5–12.5	14.9
6	Mannose	Y = 2.3615X + 3.3531 (R^2^ = 0.9991)	2.5–12.5	16.2
7	Galacturonic acid	Y = 2.1277X − 1.7009 (R^2^ = 0.9998)	1.25–12.5	25.9
8	Glucuronic acid	Y = 2.9729X − 0.4388 (R^2^ = 0.9995)	0–12.5	26.8

## Data Availability

Data are contained within the article.

## Sample Availability

Samples of the compounds are not available from the authors.
